# Correction: Active PLK1-driven metastasis is amplified by TGF-β signaling that forms a positive feedback loop in non-small cell lung cancer

**DOI:** 10.1038/s41388-019-1049-2

**Published:** 2019-10-08

**Authors:** Sol-Bi Shin, Hay-Ran Jang, Rong Xu, Jae-Yeon Won, Hyungshin Yim

**Affiliations:** Department of Pharmacy, College of Pharmacy, Institute of Pharmaceutical Science and Technology, Hanyang University, Ansan, Gyeonggi-do Korea

**Keywords:** Oncogenes, Prognostic markers

**Correction to: Oncogene**

10.1038/s41388-019-1023-z

This article was originally published under Springer Nature’s License to Publish, but has now been made available under a CC BY 4.0 license. The PDF and HTML versions of the paper have been modified accordingly.

